# Social disparities in neighborhood flood exposure in 44,698 urban neighborhoods in Latin America

**DOI:** 10.1038/s44284-025-00203-3

**Published:** 2025-02-10

**Authors:** Josiah L. Kephart, Usama Bilal, Nelson Gouveia, Olga Lucia Sarmiento, Emily Shingara, Karla Rangel Moreno, Maryia Bakhtsiyarava, Juan Pablo Rodriguez, Salvador Ayala, Gabriel Carrasco-Escobar, Ana V. Diez Roux, the SALURBAL Group, Josiah L. Kephart, Usama Bilal, Nelson Gouveia, Olga Lucia Sarmiento, Karla Rangel Moreno, Maryia Bakhtsiyarava, Juan Pablo Rodriguez, Salvador Ayala, Gabriel Carrasco-Escobar, Ana V. Diez Roux

**Affiliations:** 1 Urban Health Collaborative, Dornsife School of Public Health, Drexel University, Philadelphia, PA, USA; 2 Department of Environmental and Occupational Health, Dornsife School of Public Health, Drexel University, Philadelphia, PA, USA; 3 Department of Epidemiology and Biostatistics, Dornsife School of Public Health, Drexel University, Philadelphia, PA, USA; 4 University of São Paulo Medical School, São Paulo, Brazil; 5 School of Medicine, Universidad de los Andes, Bogotá, Colombia; 6 Instituto Nacional de Salud Pública, Cuernavaca, Mexico; 7 Institute of Transportation Studies, University of California, Berkeley, Berkeley, CA, USA; 8 Department of Civil and Environmental Engineering, Universidad de los Andes, Bogotá, Colombia; 9 Instituto de Salud Poblacional, Facultad de Medicina, Universidad de Chile, Santiago, Chile; 10 Health Innovation Laboratory, Alexander von Humboldt Institute of Tropical Medicine, Universidad Peruana Cayetano Heredia, Lima, Peru

## Abstract

Climate change is expected to greatly increase exposure to flooding, particularly among urban populations in low- and middle-income countries. Here we used daily flood data (2000–2018) to describe socioeconomic disparities in flooding on the basis of neighborhood educational attainment, comparing disparities across and within cities. We used multilevel models to examine disparities in area flooding by city- and neighborhood-level factors, including 44,698 neighborhoods in 276 cities from eight countries with a total of 223 million residents and 117 distinct flood events. One in 4 of residents of neighborhoods in the lowest quintile of educational attainment were exposed to flooding, compared with 1 in 20 residents of neighborhoods in the highest quintile of educational attainment. Neighborhoods experiencing more flooding included those with lower educational attainment, that were coastal, less dense, further from the city center and greener, and that had steeper slopes. We show large social disparities in neighborhood flooding within Latin American cities. Policymakers must prioritize flood adaptation and recovery efforts in neighborhoods with lower socioeconomic status.

Floods are the most frequently occurring climate-related disaster, affecting more people than any other disaster type^1^. Climate change is projected to increase the frequency, intensity and extent of major flood events through rising sea levels, faster snowmelt and a greater frequency of storms with extreme precipitation^2–5^. Increasing levels of urbanization have exacerbated flood hazards in densely populated areas by replacing natural rainwater sinks and watersheds that absorb precipitation with impervious surfaces that increase the amount of rainwater runoff directed toward streams^6^. The combined forces of climate change and urbanization have led to rapid growth in the proportion of the global population that lives in flood-prone areas^4,6^. Between 2000 and 2015, the global population affected by floods grew by 58–86 million, an alarming increase of 20–24% within just 16 years^4^.

Increases in population exposure to floods are unequally distributed between countries and are projected to disproportionately impact residents of low- and middle-income countries^4,7^. Within high-income countries, marginalized communities experience greater flood hazards^8–10^ and greater susceptibilities to the health, social and economic impacts of floods^10,11^. However, little is known about how flood hazards are distributed along social gradients in highly urbanized Latin America^12,13^, where wide social, health and environmental inequalities are common^14–16^ and patterns of neighborhood segregation and environmental disparities often differ from those observed in high-income countries^16–18^. Furthermore, little is known about what features of the urban environment are associated with differences in flood hazards between and within the cities of Latin America^12^. This lack of knowledge on the interplay between floods, population risk, social disparities and the urban environment is a barrier to climate adaptation planning across Latin America^12,19^, where 80% of the population lives in highly unequal urban areas^20^ and climate-related flooding is expected to increase in the coming decades^2,5,21,22^. There is a critical need to examine the linkages between population flood hazards, social disparities and the urban environment to inform flood adaptation efforts that protect public health and decrease inequality across urban Latin America^23^.

To address these gaps, we described neighborhood socioeconomic disparities in exposure to recent floods and urban characteristics associated with flooding within 276 Latin American cities diverse in climate, socioeconomic factors and features of the urban environment.

## Results

### Study neighborhoods, population and urban characteristics

**Fig. 1:**
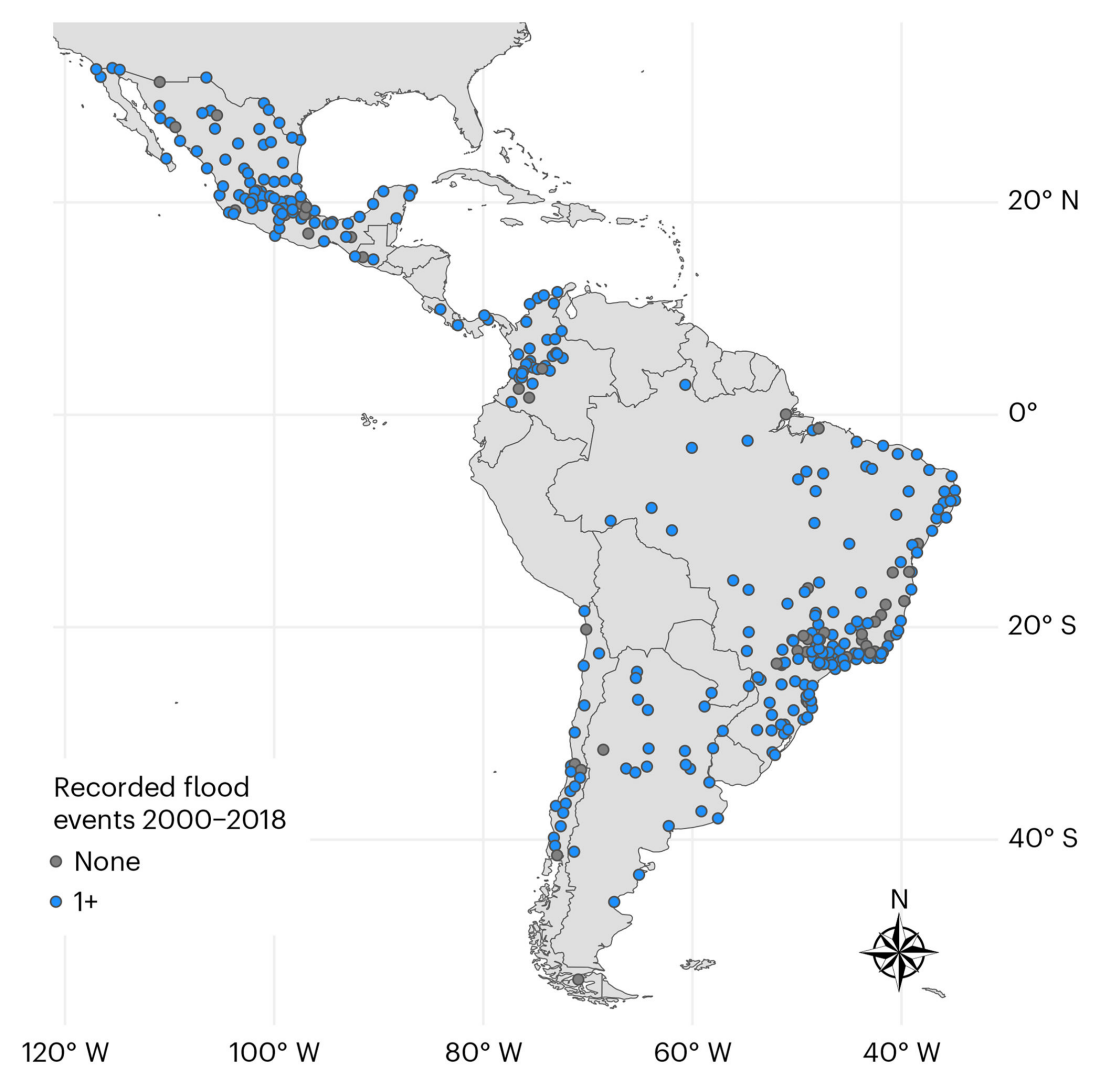
Study cities with recorded floods in the GFD from 2000 to 2018. A total of 276 cities had recorded floods and were included in the analysis (blue color); 50 cities had no recorded floods and were excluded (dark-gray color).

We examined 44,698 neighborhoods in 276 cities with a total of 117 recorded flood events in Argentina, Brazil, Chile, Colombia, Costa Rica, Guatemala, Mexico and Panama (Table 1 and Supplementary Dataset 1). The geographic locations of observed cities are shown in Fig. 1, where study cities (all with recorded flood events) are represented by blue points while excluded cities without recorded flood events (*n* = 50) are indicated as dark-gray points. Neighborhoods in Brazil had the lowest median population educational attainment (67.6% of adults aged 25+ years completed primary school, interquartile range (IQR 14.9), while neighborhoods in Mexico (91.8%, IQR 8.7) had the highest median educational attainment. Neighborhoods in Colombia had the highest population density (11.0 (IQR 15.8) thousand residents per square kilometer), while neighborhoods in Brazil were the least dense (4.1, IQR 7.5). Neighborhood and city population and characteristics are described overall and by country in Table 1.

### Neighborhood flood population exposures and education

There were 117 unique floods across the 276 study cities between 2000 and 2018. Large-scale floods often affected more than one city or country. The number of flood events ranged from 12 floods in Chile to 62 floods in Brazil (Table 2). Supplementary Dataset 1 shows the number of flood events by individual city. Of the 228.3 million residents residing in study cities as of the last census, 38.1 million people (16.7% of residents) lived in neighborhoods that flooded at least once between 2000 and 2018 (Table 2). Supplementary Fig. 2 shows neighborhood flooding within two select cities: the metropolitan area of Buenos Aires, Argentina, and Medellín, Colombia. We observed large differences by country in the percentage of urban residents in neighborhoods with flooding. While 24.0% of residents of Brazilian cities and 19.5% of residents of Colombian cities lived in neighborhoods with flooding, just 4.3% of residents in Central American cities lived in flooded neighborhoods (Table 2).

**Fig. 2:**
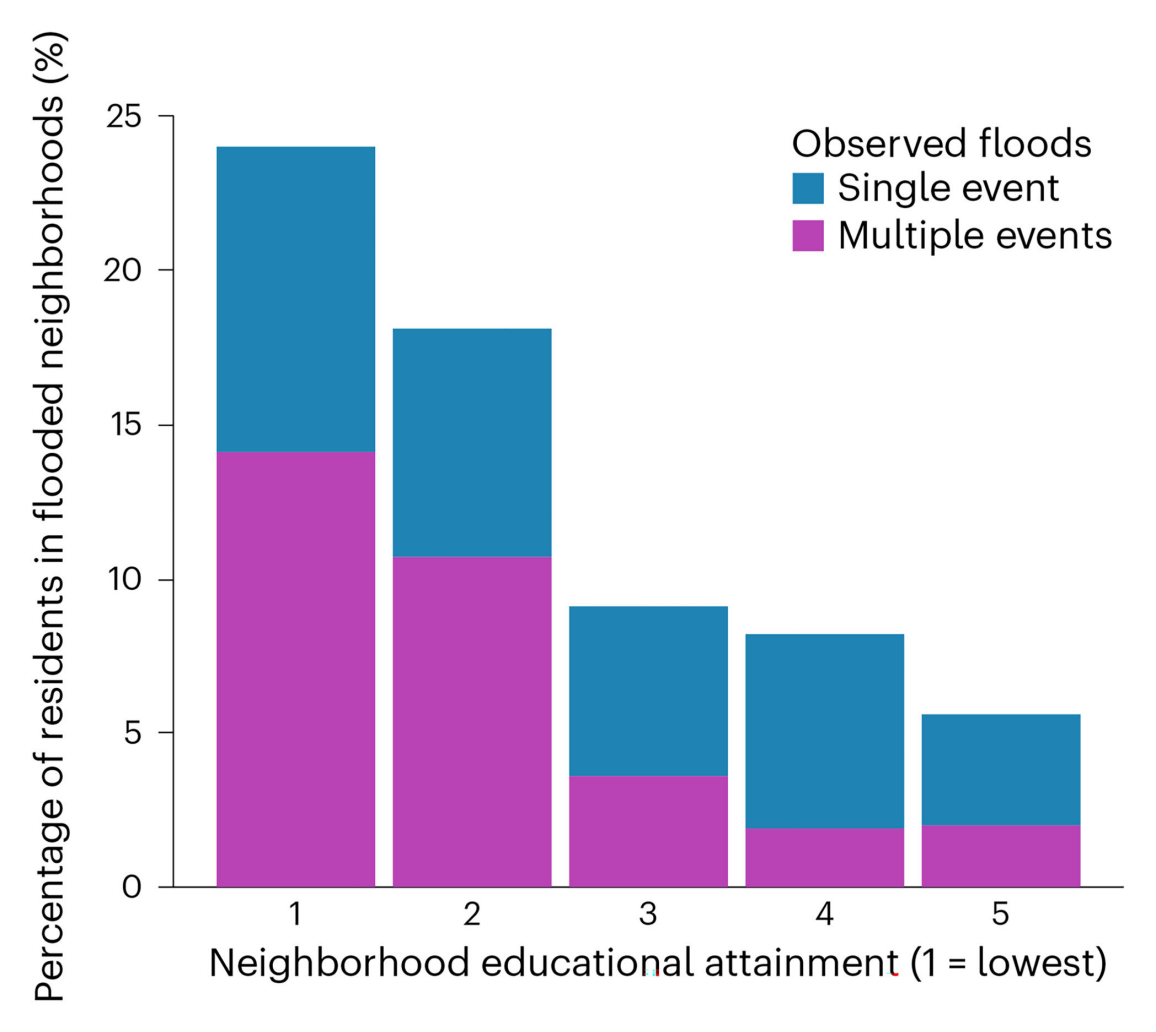
Percentage of total residents experiencing flooding in their neighborhoods. The data represent residents in 276 Latin American cities with recorded flood events between 2000 and 2018, by neighborhood education attainment (percentage of population at least 25 years of age who completed primary education) quintile.

We also observed linear associations between greater flood hazard and lower neighborhood educational attainment. Across the distribution of 44,698 study neighborhoods, 24.0% of residents of neighborhoods in the lowest quintile of education attainment lived in flooded neighborhoods, while only 5.6% of residents of the highest educational quintile neighborhoods lived in neighborhoods with flooding (Table 2 and Fig. 2). Across study cities, residents of the lowest-education neighborhoods were 4.3 times more likely to live in neighborhoods with flooding than residents of the highest-education neighborhoods. We also examined the relationship between neighborhood education and two or more neighborhood floods. The association of higher neighborhood education with lower probability of neighborhood flooding was consistent across all education quintiles and when comparing single (Table 2) and multiple floods (Fig. 2).

When we examined within-city disparities in flooding in each of 276 cities, disparities by neighborhood education were even more pronounced (Table 3). Across all 276 cities, 80.1% of cities showed educational disparities in flooding with higher prevalence of flooding in neighborhoods of lower education. The median slope index of inequality (SII) for flooding was 8.3, indicating that the prevalence of flooding was 8.3 times higher in neighborhoods of the lowest versus highest educational quintile. These disparities were very heterogeneous, with 25% of cities having an SII at or below 1.3 (that is, the prevalence of flooding was 1.3 times higher in neighborhoods of the lowest versus highest educational quintile) and 25% of cities having an SII at or above 100 (that is, the prevalence of flooding was 100 times higher in neighborhoods of the lowest versus highest educational quintile). This overall pattern of substantially higher odds of flooding in lowest versus highest education neighborhoods within cities was consistent across all countries, although the median SII for Mexican cities was substantially higher than for other countries (25.4 versus 3.0–8.6).

In many cities, the relative risk (RR) was greater than 100, indicating that floods occurred almost exclusively in lower-education neighborhoods. When stratifying cities by size (quintile of population), we found more extreme results in smaller cities.

### Neighborhood flooding and the urban environment

In Table 4, we present associations between neighborhood flooding and features of the urban environment. In multilevel models adjusting for both neighborhood and city characteristics simultaneously, higher neighborhood population density (odds ratio (OR) 0.47 per unit higher *z* score, 95% confidence interval (CI) 0.41–0.52) and intersection density (OR 0.71 per unit higher *z* score, 95% CI 0.64–0.77) were associated with lower odds of flooding. Coastal neighborhoods were substantially more likely to experience flooding than noncoastal neighborhoods (OR 46.24, 95% CI 38.30–55.82). We found that neighborhoods that were greener (OR 1.43 per unit higher *z* score, 95% CI 1.32–1.55) and more distant from the city center (OR 1.19 per unit higher *z* score, 95% CI 1.13–1.25) were more likely to experience flooding compared with less green, central neighborhoods. In this same model, higher altitude and flatter slope were also associated with higher odds of neighborhood flooding. In our sensitivity analysis restricting these models to the 196 cities with two or more flood events, we found nearly identical associations, suggesting that flood patterns within cities are reasonably captured by as few as one recorded flood event (Supplementary Table 2). In our sensitivity analysis using an alternative metric of greenspace (greenspace as percentage of neighborhood land area, replacing the normalized difference vegetation index (NDVI)), we found qualitatively similar findings (Supplementary Table 3).

Our descriptive findings of educational disparities in neighborhood flooding were consistent even after adjusting for urban features. After simultaneously adjusting for a suite of city- and neighborhood-level urban and natural features, neighborhoods with higher education had lower odds of flooding than neighborhoods with lower education (OR 0.81 per unit higher educational attainment *z* score, 95% CI 0.75–0.86). However, we did not find an association between city-level education and flood hazard.

## Discussion

We conducted a descriptive analysis at high spatial resolution of 117 floods across nearly 45,000 neighborhoods of 276 cities in eight Latin American countries, representing 228 million residents. Our study provides four key findings. First, 17% of the 228 million residents of these cities lived in neighborhoods with at least one flood during the study period. Second, there are substantial disparities in flood exposure by education attainment across Latin American cities. Across the region, residents of neighborhoods in the lowest education quintile were 4.3 times more likely to live in a neighborhood that had flooded than residents of neighborhoods in the highest education quintile. Third, there are massive disparities within cities of which neighborhoods did and did not experience flooding. Within each of 276 cities, the median RR of neighborhood flooding was eight times higher in neighborhoods with the lowest education levels than in those with the highest education levels within the same city. Overall, 80% of cities demonstrated educational inequities in flood exposure, a pattern consistent across all study countries. Fourth, among Latin American cities, coastal neighborhoods and neighborhoods that are less dense, greener and peripheral to the city center were more likely to flood.

Our study identified consistent patterns of social inequities in neighborhood flooding within cities across Latin America. We are unaware of other studies that report social disparities in floods in Latin America at the neighborhood level, yet our findings corroborate other studies at higher geographic levels. Rentschler et al. examined flood exposure and poverty in 188 countries, with some subnational analyses at the department or state level, finding that, as of 2020, 89% of the world’s flood-exposed people lived in low- and middle-income countries^7^. Within Latin America, Rasch et al. conducted a national analysis of Brazil at the municipal level, finding that differences in income across municipalities predict flood hazards^24^. While there is a growing recognition of environmental injustices in flood exposure at the national and subnational levels^8^, we provide initial evidence of detrimental social inequities in neighborhood flooding within cities in Latin America. In the median city, we found that the lowest-education neighborhoods have eight times greater RR of flooding than the highest-education neighborhoods and that these inequities persist (but vary) across all study countries. We emphasize that this analysis is descriptive and does not provide causal evidence on whether flooding impacts neighborhood socioeconomic status (SES), or whether individuals of lower SES disproportionately move to neighborhoods that flood (or both). Moreover, we found that neighborhoods with lower density (population and intersection), with more vegetation and that are peripheral to the urban core face higher odds of flooding. This may reflect patterns of social segregation and urban infrastructure in Latin America, where, in many cities, communities on the urban periphery are more likely to experience lower SES^17^ and inferior municipal services^25^.

Our study should be interpreted in the context of some limitations. Although we examined neighborhood flooding at an exceptionally fine spatial scale, we were unable to examine or draw conclusions regarding personal exposure to a flood event. Nevertheless, flooding is directly and indirectly disruptive to communities even beyond injury to specific individual people or houses. Our focus was on describing disparities across neighborhoods using neighborhood education as a proxy for neighborhood socioeconomic characteristics. These analyses of neighborhood inequities are highly relevant to the spatial targeting of protective interventions or policies. The use of neighborhood education as a proxy of neighborhood SES has its limitations and may not fully capture neighborhood inequities. Neighborhood educational attainment has been demonstrated to be a key driver of disparities in life expectancy^26^ in Latin America. However, other facets of neighborhood SES, as well as neighborhood racial or ethnic segregation^27,28^, are also important and may better capture inequities than neighborhood education alone. An important assumption of our analyses is that the neighborhood SES measure that we use is relatively stable over time. This is supported by additional analyses we conducted of the correlation of the same measure of educational attainment (percentage of adults who completed high school) at a more aggregated, subcity (that is, municipality) level across the two most recent censuses (these data were available only at the subcity, not neighborhood, level). We found a Pearson correlation coefficient of 0.96 between educational attainment in the same subcity area longitudinally across consecutive census surveys, suggesting a high level of consistency in areas that have higher versus lower educational attainment over the study period.

Although the Global Flood Database (GFD) provides high-spatial-resolution estimates of flooding events, particularly for low- and middle-income countries, it represents 29% of floods events reported in the Dartmouth Flood Observatory (DFO) (and 28% within our eight study countries). Of note, nearly half (47%) of excluded events were excluded because no flooding beyond permanent water was detected at 250 m resolution. This could imply that hyperlocal, small-scale flooding events were more likely to be excluded. If these tend to be more concentrated in lower-SES neighborhoods (as suggested by other research^25^), our results may underestimate associations of lower neighborhood SES with more flooding. Another major reason for exclusion of the flood event (23% of excluded events) was cloud cover. It is unlikely that this type of exclusion (or exclusions for other miscellaneous reasons) systematically excluded flood events that were different in their neighborhood SES distribution than those included, and therefore are unlikely to have substantially impacted our findings. Nevertheless, more complete flood hazard data at fine spatial resolution are needed to better understand the spatial and social distributions of flood hazards. We also emphasize that, although the GFD data can provide insight into withincity distributions of flood hazards, they should not be used to derive estimates of the prevalence of urban flooding in Latin America given the low proportion of DFO events included and the possible limitations of the DFO data. As a sensitivity analysis, we included only cities with at least two recorded floods and found similar findings, suggesting that overall patterns of social disparities in flood exposure are somewhat consistent across cities with one versus multiple observed flood events.

Furthermore, we found an association between higher odds of flooding and higher greenness (both NDVI or, as a sensitivity analysis, percentage of area that is greenspace) in models adjusted for population and intersection density, slope, altitude and distance from city center. This may reflect higher levels of natural vegetation in flood plains, wetlands and other flood-prone areas as greater concentration of water correlates with both flood hazard and vegetation. Vegetation is particularly effective at absorbing rainwater at the point where precipitation falls, reducing stormwater runoff into waterways. However, urban centers are often built along waterways that may be some distance from where rainfall occurs, and localized urban vegetation may be insufficient to prevent localized flooding from watershed-level precipitation events. We were unable to include other indicators of the ecological and geological capacity for water accumulation or for density of rivers and tributaries. Further research that incorporates additional ecological information and longitudinal analyses may help shed light on the potentially bidirectional linkages between vegetation and neighborhood flood hazard, and how these linkages vary by climate.

Our findings reinforce the importance of prioritizing the needs of socially marginalized communities in environmental risk management and climate adaptation policies^29^. We found substantial within-city disparities in flood exposure and higher flood hazards among geographically peripheral communities, underscoring the need for urban policymakers to allocate resources toward mitigating the human impacts of flooding and not only economic impacts. While environmental inequities between the Global North and Global South are substantial, these disparities can also be found at a local level.

Our analysis integrated high-quality satellite-derived estimates of large, historical flood events from 2000 to 2018 with harmonized census records from 8 countries to examine social disparities in flood exposure across 228 million residents of 44,698 neighborhoods in 276 Latin American cities. We found consistent intraurban disparities in flood exposure, with less educated, coastal and peripheral communities often more vulnerable. These patterns persisted across all study countries. As climate change increases human exposure to floods, our findings highlight the urgent need for policymakers to implement environmental and climate adaptation actions that protect marginalized communities^13^.

## Methods

### Study setting

We conducted this study as part of the *Salud Urbana en América Latina* (SALURBAL) project. This international scientific collaboration has compiled and harmonized data on social, environmental and health characteristics for all cities of 100,000 residents or more in 2010 in 11 Latin American countries (*n* = 371)^30^. In SALURBAL, cities are defined as clusters of administrative units (that is, municipalities) or single administrative units encompassing the visually apparent built-up area of urban agglomerations as identified using satellite imagery^31^ and include a diverse set of cities, from small cities to megacities.

We excluded cities in El Salvador, Nicaragua and Peru owing to the lack of available data at the neighborhood level. Because this study focuses on within-city disparities in flood hazards, we excluded 50 cities without any recorded flood events during the study period within city boundaries. Accordingly, we examined neighborhoods in 276 cities in Argentina, Brazil, Chile, Colombia, Costa Rica, Guatemala, Mexico and Panama. Neighborhood administrative units varied in name and official definition by country. We used the country-specific, small-area administrative units most analogous to US census tracts, henceforth referred to as neighborhoods. Across countries, these neighborhoods had a median population of 2,137 and a median area of 0.36 km^2^ (equivalent to a square with 0.6 km sides). Detailed information on the administrative units used for each country is available in Supplementary Table 1.

### Neighborhood flood exposures

Estimates of recent flooding were extracted from the GFD^4^. The GFD examines flood events identified by the DFO. The DFO monitors and compiles generally large-media-coverage flood events from news sources from every country in the world, synthesizing information from news reports to estimate flood start and end dates and the geographical area affected. For floods identified in the DFO database, the GFD team analyzed satellite imagery to estimate the extent and duration of individual flood events at daily, 250-m resolution extending the geographic area of interest to include all watersheds intersecting with DFO records of flood-affected areas. The final GFD product is a database of global raster datasets showing the maximum spatial extent of flooding at 250-m resolution for 913 large flood events worldwide from 2000 to 2018 (ref. 4). A total of 273 flood events compiled by the DFO affected any part of SALURBAL cities between 2000 and 2018, and a subset of 117 (43%) were included in the GFD and, therefore, this analysis. Globally, the reasons for flood events in the DFO not being included in the GFD database were a lack of detectable inundation at a 250-m resolution within the geographic areas reported as flood-affected by news reports (47% of excluded events), persistent cloud cover preventing satellite observation (23% of excluded events) or other/unknown causes (30%). Further details on the GFD data sources and methods have been previously published^4^. To link flood frequency data to city and neighborhood data, we overlayed floods events in the GFD with SALURBAL city and neighborhood boundaries. We considered a neighborhood to have flooded if flooding occurred anywhere within the neighborhood boundary. The resulting analysis dataset was the cumulative number of recorded, distinct flood events between 2000 and 2018 for each neighborhood in 276 Latin American cities.

### Neighborhood and city characteristics

We used data on neighborhood characteristics and population compiled from national census bureaus and other sources by the SALURBAL project^31^. We used the most recent available census for each country with census years ranging from 2002 to 2018. Information on the year of each census used is available in Supplementary Table 1. We compiled neighborhood and city educational attainment (percentage of the population aged 25 years or older who completed primary education or above) from censuses, as this was the most relevant socioeconomic variable available across all eight national census surveys. We used neighborhood education as a proxy for neighborhood SES conditions. This is a commonly used proxy for neighborhood SES and has been used alone or in combination with other neighborhood metrics in other analyses^17,26,32^. Within Latin America, educational attainment has been shown to be a key driver of disparities in life expectancy^26^. At both the neighborhood and city levels, the SALURBAL project previously estimated population density (population divided by built-up area)^31^, intersection density (density of the set of nodes with more than one street emanating from them per square kilometer of built-up area)^31^, area median greenness measured by the NDVI^33^ and, as a sensitivity analysis, area greenspace as a percentage of total land (that is, nonwater) area. We defined neighborhoods as coastal if their spatial boundaries were within 1 km of an ocean coastline. For each neighborhood, we calculated distance from the city center as the Euclidean distance (km) between the neighborhood centroid and, generally, city hall. Neighborhood mean elevation and mean slope were estimated using a well-established 30-m-resolution topography model^34^, excluding permanent water. At the city level, we also used population estimates from national censuses and statistical agencies^14^, gross domestic product (GDP) per capita (computed as purchasing power parities in constant 2011 international US dollars of each city in 2015 derived from regional estimates)^35^, and Köppen climate zone (first level)^36^. Supplementary Appendix 1 details the creation of urban environment variables.

### Statistical analysis

We calculated summary statistics of neighborhood-level exposures to flooding by country and city and stratified by neighborhood educational attainment overall and by country. We estimated the total population in cities with recorded flooding and the number and percentage of the total city population residing in neighborhoods that had experienced flooding. We grouped neighborhoods into quintiles of neighborhood educational attainment (percentage of the population aged 25 years or older who completed primary education or above) based on the full sample of study neighborhoods. Due to the small number of cities represented by each country in Central America, we pooled the cities of Costa Rica (*n* = 1 city), Guatemala (*n* = 1) and Panama (*n* = 3) into a single grouping for all analyses.

To describe magnitudes of within-city disparities in flooding, we used a city-specific Poisson model with neighborhood flooding (yes/no) as the outcome and city-specific quintiles of education as the exposure, operationalized as ordinal and rescaled from 0 to 1. This makes the interpretation of the education coefficient analogous to a SII: the RR of flooding comparing lowest versus highest levels of education, while considering the entire distribution of education. A city-specific RR of >1 signifies higher prevalence of flooding in lower-education neighborhoods.

To examine associations of city and neighborhood characteristics with flooding, we used multilevel logistic models with a random intercept for each city and fixed effect for country. All neighborhood variables were operationalized as *z* scores of the overall distribution of 44,698 neighborhoods for each respective variable, except for coastal neighborhood (yes/no), altitude (meters above sea level) and mean slope (degrees). Similarly, city-level variables were operationalized as *z* scores of the overall distribution of 276 cities for each variable, with the exception of climate zone (categorical: temperate, arid or tropical). We first conducted a univariable analysis of each independent variable and the dependent variable of neighborhood flooding. We then assessed all variables for collinearity using Spearman correlation coefficients (Supplementary Fig. 1). No covariates had correlation coefficients greater than 0.57 (city greenness versus neighborhood greenness), so we modeled all neighborhood- and city-level predictors concurrently as a single multivariable model. As a sensitivity analysis, we repeated all models above but leveraged the same multivariable modeling approach but using only the 196 cities with at least two floodings events. As an additional sensitivity analysis, we used an alternate measure of greenspace (greenspace as percentage of neighborhood land area), which replaced NDVI in the models. Reported 95% CIs are from Wald two-tailed tests. The assumptions of these tests were determined to have been met plotting model residuals for normality of residuals and heteroskedasticity. Data processing and analyses were conducted in R version 4.4.1 (ref. 37), and multilevel models were performed using the lme4 package^38^.

### Ethics and inclusion statement

All co-authors are co-investigators and/or trainees in the SALURBAL Group, an international collaboration of researchers from institutions across the Americas. A majority of authors are based at institutions within the study region (that is, Latin American urban areas). All co-authors have been involved in all steps of study design and implementation since the start of study formation through manuscript development and the decision to submit for publication.

### Reporting summary

Further information on research design is available in the Nature Portfolio Reporting Summary linked to this article.

## Supplementary Material

Reporting Summary

Supplementary Dataset 1

Supplementary Tables 1–3, Figs. 1 and 2 and Appendix 1.

## Figures and Tables

**Table 1:** Population and urban environment characteristics of 44,698 study neighborhoods in 276 Latin American cities with recorded flood events in the GFD from 2000 to 2018, overall and by country. Results presented as number (*n*) and percent or mean (standard deviation)

	Total	Argentina	Brazil	Central America^a^	Chile	Colombia	Mexico
**Neighborhood level**							
*n* of neighborhoods	44,698	2,002	3,660	4,929	1,824	2,907	29,376
Population density^b^	8.1 (11.0)	8.7 (9.8)	6.3 (7.1)	11.1 (24.8)	5.9 (5.0)	12.9 (11.9)	7.5 (6.9)
Educational attainment (% primary school or less)	86.3 (13.6)	89.4 (7.9)	67.9 (11.8)	73.9 (22.0)	88.9 (9.5)	88.7 (14.5)	90.5 (8.0)
Intersection density^c^	150 (172)	81 (43)	89 (57)	101 (112)	135 (101)	251 (544)	162 (100)
Greenness (NDVI)^d^	0.47 (0.20)	0.46 (0.20)	0.57 (0.19)	0.60 (0.19)	0.46 (0.22)	0.57 (0.20)	0.42 (0.18)
Distance from city center (km)	12.7 (13.8)	21.2 (19.2)	14.9 (14.5)	11.8 (8.1)	9.2 (12.0)	9.7 (12.4)	12.5 (14.1)
*n* (%) of coastal neighborhoods	2,244 (5.0%)	149 (7.4%)	592 (16.2%)	160 (3.2%)	358 (19.6%)	182 (6.3%)	803 (2.7%)
Altitude (m)	1,063 (921)	134 (287)	409 (369)	1,409 (675)	218 (429)	1,163 (992)	1,253 (943)
Slope (°)	4.4 (3.8)	2.8 (2.0)	5.2 (3.4)	7.0 (5.3)	5.0 (4.4)	6.0 (5.7)	3.8 (3.0)
**City level**							
*n* of cities	276	21	124	5	16	29	81
City population (×1,000)	721 (2,036)	1,023 (3,078)	715 (2,021)	1,130 (1,018)	228 (158)	759 (1,479)	711 (2,168)
GDP per capita (US$, ×1,000)	15.5 (11.3)	17.4 (7.2)	15.2 (8.1)	16.9 (7.4)	21.2 (18.3)	10.3 (3.8)	16.1 (15.5)
Population density^b^	7.1 (3.8)	5.6 (1.3)	6.1 (2.1)	7.5 (2.7)	7.0 (1.4)	15.7 (5.1)	5.9 (1.5)
Education (% primary school or less)	74.1 (10.0)	79.6 (2.8)	65.6 (6.1)	86.6 (6.1)	89.5 (2.5)	83.9 (3.7)	78.2 (7.0)
Intersection density^c^	92.3 (25.3)	85.1 (16.3)	84.4 (19.2)	60.5 (23.7)	125.1 (15.1)	117.6 (31.1)	92.9 (23.4)
Greenness (NDVI)	0.56 (0.12)	0.52 (0.11)	0.60 (0.06)	0.69 (0.06)	0.42 (0.20)	0.63 (0.07)	0.50 (0.14)

a Central America grouping includes urban neighborhoods in Costa Rica (*n* = 1 city), Guatemala (*n* = 1) and Panama (*n* = 3).

b Thousands of residents per square kilometer.

c Street intersections per square kilometer.

d Median normalized vegetation index within the neighborhood or city.

**Table 2:** Neighborhood and population exposures to flood events, by neighborhood education attainment and country in 44,698 neighborhoods in 276 cities in eight Latin American countries from 2000 to 2018

	Total neighborhoods	Number of unique flood events^b^	City-specific percentage of neighborhoods with flooding: median (P10, P90)^c^	Total population (millions)	Population in flooded neighborhoods (millions)	Percentage of total population in flooded neighborhoods
**Overall**	44,698	117	14% (1, 56)	228.3	38.1	16.7%
**By country grouping**						
Argentina	2,002	43	31% (7, 66)	23.2	2.8	12.1%
Brazil	3,660	62	23% (8, 75)	101.0	24.4	24.0%
Central American^a^	4,929	17	2% (1, 6)	6.2	0.3	4.3%
Chile	1,824	12	5% (2, 26)	5.1	0.4	8.2%
Colombia	2,907	23	17% (3, 37)	22.4	4.4	19.5%
Mexico	29,376	31	2% (1, 11)	70.0	5.9	8.4%
**By neighborhood educational attainment**						
1st quintile (lowest)	8,940	–	–	97.6	23.5	24.0%
2nd	8,940	–	–	42.1	7.6	18.2%
3rd	8,940	–	–	34.4	3.1	9.1%
4th	8,939	–	–	32.7	2.7	8.2%
5th quintile (highest)	8,939	–	–	21.5	1.2	5.6%

a Includes cities in the countries of Costa Rica (*n* = 1 city), Guatemala (*n* = 1) and Panama (*n* = 3).

b The number of unique flood events overall does not equal the sum of unique flood events by country because some floods affected multiple countries (as well as multiple cities).

c P10 = 10th percentile; P90 = 90th percentile.

**Table 3:** Within-city disparities in flood exposure by neighborhood educational attainment within 276 cities in eight countries of Latin America that experienced flooding between 2000 and 2018

	City-specific slope indicesof inequality of flooding in lowest versus highest education neighborhoods			Percentage of cities with higher RR of flooding in lowest versus highest education quintile neighborhoods (SII >1)
	25th percentile	Median	75th percentile	
**Overall**	1.3	8.3	>100	80.1%
**By country grouping**				
Argentina	1.6	5.6	27.5	85.7%
Brazil	1.0	4.0	>100	73.4%
Central America^a^	2.0	5.0	9.4	80.0%
Chile	1.8	8.6	>100	81.2%
Colombia	1.7	3.0	30.9	86.2%
Mexico	2.3	25.4	>100	86.4%
**By city population size**				
1st quintile (smallest)	2.5	25.2	>100	82.1%
2nd	1.2	8.7	>100	78.2%
3rd	2.0	9.6	>100	85.5%
4th	1.2	13.6	>100	80.0%
5th quintile (largest)	1.0	2.13	8.2	74.5%

a Includes cities in the countries of Costa Rica (*n* = 1 city), Guatemala (*n* = 1) and Panama (*n* = 3).

**Table 4:** ORs of neighborhood flooding associated with a one-standard-deviation higher value for neighborhood- and city-level features in 44,698 neighborhoods in 276 cities in eight Latin American countries from 2000 to 2018

		Univariable			Multivariable (all)	
		Estimate	95% CI		Estimate	95% CI
Neighborhood level	Population density	**0.24**	0.22, 0.27		**0.47**	0.41, 0.52
	Education	**0.67**	0.63, 0.70		**0.81**	0.76, 0.86
	Intersection density	**0.31**	0.28, 0.34		**0.71**	0.65, 0.78
	Greenness	**1.96**	1.86, 2.06		**1.43**	1.32, 1.55
	Distance from city center	**1.52**	1.40, 1.58		**1.19**	1.13, 1.25
	Coastal (<1 km)	**33.4**	28.1, 39.6		**46.6**	38.6, 56.3
	Altitude	0.88	0.74, 1.04		**1.39**	1.16, 1.66
	Slope	**1.09**	1.04, 1.14		**0.79**	0.75, 0.84
	Population size	**0.86**	0.76, 0.99		0.95	0.83, 1.09
	Population density	0.94	0.74, 1.18		0.93	0.72, 1.19
	Education	0.87	0.66, 1.13		0.95	0.72, 1.25
City level	Intersection density	**0.81**	0.68, 0.97		0.99	0.83, 1.19
	Greenness/vegetation	**1.31**	1.11, 1.55		0.97	0.78, 1.20
	GDP	0.98	0.84, 1.15		1.03	0.87, 1.20
	Climate					
	- Temperate	1 (reference)			1 (reference)	
	- Arid	0.68	0.43, 1.08		0.62	0.36, 1.07
	- Tropical	**1.75**	1.21, 2.53		1.15	0.78, 1.70

All independent variables have been *z*-transformed using the distribution of all study neighborhoods, except for proximity to coast, which is binary (yes/no <1 km from coast), and climate zone (categorical). Estimates in bold font represent effect sizes where 95% CI does not cross unity.

## Data Availability

Historical flood event data were downloaded from the publicly available GFD at https://developers.google.com/earth-engine/datasets/catalog/GLOBAL_FLOOD_DB_MODIS_EVENTS_V1. Population and census data for Brazil, Chile and Mexico were downloaded from publicly available repositories from statistical agencies in each country. Population and census data for Argentina, Costa Rica, El Salvador, Guatemala, Panama and Peru were obtained directly from statistical agencies in each country. A link to these agency websites can be accessed via https://drexel.edu/lac/data-evidence/data-acknowledgements/. Urban boundaries and features obtained from the SALURBAL-Climate project are freely available at https://data.lacurbanhealth.org/. Please contact the corresponding author about access to any urban features that are not yet published on the SALURBAL-Climate portal.

## Code availability

Code for the main and secondary analyses is publicly available via GitHub at https://github.com/Drexel-UHC/MS218A_LA_flood_disparities.
