# Supplementary material for: Social disparities in neighborhood flood exposure in 44,698 urban neighborhoods in Latin America
Source: Nat Cities. Author manuscript; Available in PMC 2025 Apr 4. (PMC7617554; doi:10.1038/s44284-025-00203-3)
Supplement: Reporting Summary [file EMS203970-supplement-Reporting_Summary.pdf]

nature portfolio

Corresponding author(s): Josiah L. Kephart, PhD

Last updated by author(s): Dec 19, 2024

Reporting Summary

Nature Portfolio wishes to improve the reproducibility of the work that we publish. This form provides structure for consistency and transparency in reporting. For further information on Nature Portfolio policies, see our Editorial Policies and the Editorial Policy Checklist.

Statistics

For all statistical analyses, confirm that the following items are present in the figure legend, table legend, main text, or Methods section.

n/a	Confirmed	
☐	☒	The exact sample size (*n*) for each experimental group/condition, given as a discrete number and unit of measurement
☐	☒	A statement on whether measurements were taken from distinct samples or whether the same sample was measured repeatedly
☐	☒	The statistical test(s) used AND whether they are one- or two-sided<br>*Only common tests should be described solely by name; describe more complex techniques in the Methods section.*
☐	☒	A description of all covariates tested
☐	☒	A description of any assumptions or corrections, such as tests of normality and adjustment for multiple comparisons
☐	☒	A full description of the statistical parameters including central tendency (e.g. means) or other basic estimates (e.g. regression coefficient) AND variation (e.g. standard deviation) or associated estimates of uncertainty (e.g. confidence intervals)
☐	☒	For null hypothesis testing, the test statistic (e.g. *F*, *t*, *r*) with confidence intervals, effect sizes, degrees of freedom and *P* value noted<br>*Give P values as exact values whenever suitable.*
☒	☐	For Bayesian analysis, information on the choice of priors and Markov chain Monte Carlo settings
☒	☐	For hierarchical and complex designs, identification of the appropriate level for tests and full reporting of outcomes
☒	☐	Estimates of effect sizes (e.g. Cohen's *d*, Pearson's *r*), indicating how they were calculated

Our web collection on statistics for biologists contains articles on many of the points above.

Software and code

Policy information about availability of computer code

Data collection	All data collection was conducted in R (version 4.4.1).
Data analysis	All analyses were performed in R (version 4.4.1) and modeling was done with the lme4 package. Analysis code is available at: https://github.com/Drexel-UHC/MS218A_LA_flood_disparities

For manuscripts utilizing custom algorithms or software that are central to the research but not yet described in published literature, software must be made available to editors and reviewers. We strongly encourage code deposition in a community repository (e.g. GitHub). See the Nature Portfolio guidelines for submitting code & software for further information.

Data

Policy information about availability of data

All manuscripts must include a data availability statement. This statement should provide the following information, where applicable:

- Accession codes, unique identifiers, or web links for publicly available datasets
- A description of any restrictions on data availability
- For clinical datasets or third party data, please ensure that the statement adheres to our policy

Historical flood event data was downloaded from the publicly available Global Flood Database at https://developers.google.com/earth-engine/datasets/catalog/GLOBAL_FLOOD_DB_MODIS_EVENTS_V1. Population and census data for Brazil, Chile, and Mexico were downloaded from publicly available repositories from statistical agencies in each country. Population, and census data for Argentina, Costa Rica, El Salvador, Guatemala, Panama and Peru were obtained directly from

statistical agencies in each country. A link to these agency websites can be accessed via https://drexel.edu/lac/data-evidence/data-acknowledgements/. Urban boundaries and features were obtained from the SALURBAL-Climate project and are freely available at https://data.lacurbanhealth.org/. Please contact the corresponding author about access to any urban features that are not yet published on the SALURBAL-Climate portal.

Research involving human participants, their data, or biological material

Policy information about studies with human participants or human data. See also policy information about sex, gender (identity/presentation), and sexual orientation and race, ethnicity and racism.

Reporting on sex and gender	Not applicable
Reporting on race, ethnicity, or other socially relevant groupings	Not applicable
Population characteristics	Not applicable
Recruitment	Not applicable
Ethics oversight	Not applicable

Note that full information on the approval of the study protocol must also be provided in the manuscript.

Field-specific reporting

Please select the one below that is the best fit for your research. If you are not sure, read the appropriate sections before making your selection.

☐ Life sciences ☒ Behavioural & social sciences ☐ Ecological, evolutionary & environmental sciences

For a reference copy of the document with all sections, see nature.com/documents/nr-reporting-summary-flat.pdf

Behavioural & social sciences study design

All studies must disclose on these points even when the disclosure is negative.

Study description	This quantitative study is a cross-sectional, ecological analysis of neighborhood level flood exposure, educational disparities in flooding, and associations with the neighborhood and city urban environments.
Research sample	Our data consists of every city (N=326 cities) with at least 100,000 residents in Argentina, Brazil, Chile, Colombia, Costa Rica, Guatemala, Mexico, and Panama. Cities were identified by the SALURBAL project. We excluded N=50 cities without recorded floods from 2000-2018, leaving N=276 cities in our main analysis. These 276 cities had a total of 228.3 million residents as of the most recent census.
Sampling strategy	We included all cities of at least 100,000 residents, as of 2010, within eight Latin American countries. This threshold was selected to capture cities from a range of population sizes, from small cities to megacities. We included the entire population, without exclusion for age, sex, or otherwise.
Data collection	We used the Global Flood Database to estimate cumulative flooding from 2000-2018 for each neighborhood. Population counts and area-level education attainment were compiled directly from governmental vital records and census agencies. The analysis was completed by the first author, who also conceptualized the study and hypotheses.
Timing	The observation period was from 2000-2018, as per availability of published flood data from the Global Flood Database.
Data exclusions	We excluded cities without recorded flood events (N=50), leaving a total of N=276 cities in the main analysis. As the study focused on within-city disparities, we were unable to observe disparities within cities with no reported flood events.
Non-participation	No participants were involved in the study.
Randomization	This study is designed to examine cross-sectional associations between historical flooding and neighborhood educational attainment. We grouped neighborhoods by quintile of educational attainment based on the full sample of study neighborhoods.

Reporting for specific materials, systems and methods

We require information from authors about some types of materials, experimental systems and methods used in many studies. Here, indicate whether each material, system or method listed is relevant to your study. If you are not sure if a list item applies to your research, read the appropriate section before selecting a response.

Materials & experimental systems

n/a	Involved in the study
☒	☐ Antibodies
☒	☐ Eukaryotic cell lines
☒	☐ Palaeontology and archaeology
☒	☐ Animals and other organisms
☒	☐ Clinical data
☒	☐ Dual use research of concern
☒	☐ Plants

Methods

n/a	Involved in the study
☒	☐ ChIP-seq
☒	☐ Flow cytometry
☒	☐ MRI-based neuroimaging

Plants

Seed stocks	*Report on the source of all seed stocks or other plant material used. If applicable, state the seed stock centre and catalogue number. If plant specimens were collected from the field, describe the collection location, date and sampling procedures.*
Novel plant genotypes	*Describe the methods by which all novel plant genotypes were produced. This includes those generated by transgenic approaches, gene editing, chemical/radiation-based mutagenesis and hybridization. For transgenic lines, describe the transformation method, the number of independent lines analyzed and the generation upon which experiments were performed. For gene-edited lines, describe the editor used, the endogenous sequence targeted for editing, the targeting guide RNA sequence (if applicable) and how the editor was applied.*
Authentication	*Describe any authentication procedures for each seed stock used or novel genotype generated. Describe any experiments used to assess the effect of a mutation and, where applicable, how potential secondary effects (e.g. second site T-DNA insertions, mosiacism, off-target gene editing) were examined.*
