# Supplementary material for: Social disparities in neighborhood flood exposure in 44,698 urban neighborhoods in Latin America
Source: Nat Cities. Author manuscript; Available in PMC 2025 Apr 4. (PMC7617554; doi:10.1038/s44284-025-00203-3)
Supplement: Supplementary Tables 1–3, Figs. 1 and 2 and Appendix 1. [file EMS203970-supplement-Supplementary_Tables_1_3__Figs__1_and_2_and_Appendix_1_.pdf]

nature cities

Supplementary information

https://doi.org/10.1038/s44284-025-00203-3

Social disparities in neighborhood flood exposure in 44,698 urban neighborhoods in Latin America

In the format provided by the authors and unedited

Table of Contents

Supplemental Table S1: Neighborhood administrative units and years of most recent available census… 2

Supplemental Table S2: Multilevel associations between flooding and urban features comparing main analysis (including all cities with one or more recorded flood events) and sensitivity analysis (including only cities with at least two recorded flood events). 3

Supplemental Table S3: Multilevel associations between flooding and urban features comparing alternative greenspace metrics in main analysis (NDVI) and sensitivity analysis (greenspace as % of neighborhood land area). 4

Supplemental Figure S1: Spearman correlation coefficients for covariates in the main model of the associations between neighborhood flooding and features of the urban environment. 5

Supplemental Figure S2: Within-city variation in neighborhood flooding in two selected cities: (a) Buenos Aires, Argentina and (b) Medellin, Colombia. Black lines represent neighborhood boundaries and colors represent number of distinct floods from 2000-2018 in the Global Flood Database. The metropolitan area of Buenos Aires, Argentina (left panel), and Medellín, Colombia (right panel)……..... 6

Appendix I: Detailed descriptions of urban environmental measures and their interpretation 7

Supplemental Table S1: Neighborhood administrative units and years of most recent available census.

Country	Administrative unit (neighborhood)	Census year
Argentina	*Fraccion Censal*	2010
Brazil	*Áreas de Ponderação*	2010
Chile	*Zona Censal*	2017
Colombia	*Sector Urbano, Clase = 1*	2018
Costa Rica	*Distrito*	2011
Guatemala	*Sector Censal*	2002
Mexico	*Área Geoestadistica Básica*	2010
Panama	*Barrio*	2010

Supplemental Table S2: Multilevel associations between flooding and urban features comparing main analysis (including all cities with one or more recorded flood events) and sensitivity analysis (including only cities with at least two recorded flood events).

		Main analysis: 1+ flood events (N=276 cities)		Sensitivity analysis: 2+ flood events (N=196 cities)	
		Estimate	95% CI	Estimate	95% CI
Neighborhood-Level	Population density*	**0.47**	0.41, 0.52	**0.46**	0.40, 0.52
	Education	**0.81**	0.76, 0.86	**0.81**	0.75, 0.87
	Intersection density	**0.71**	0.65, 0.78	**0.78**	0.71, 0.85
	Greenness	**1.43**	1.32, 1.55	**1.52**	1.40, 1.66
	Distance from city center	**1.19**	1.13, 1.25	**1.16**	1.10, 1.23
	Coastal (<1 km)	**46.6**	38.6, 56.3	**62.4**	49.9, 78.0
	Altitude	**1.39**	1.16, 1.66	**1.59**	1.29, 1.96
	Slope	**0.79**	0.75, 0.84	**0.76**	0.71, 0.82
City-level	Population size	0.95	0.83, 1.09	0.92	0.77, 1.10
	Population density	0.93	0.72, 1.19	0.84	0.62, 1.15
	Education	0.95	0.72, 1.25	0.97	0.71, 1.32
	Intersection density	0.99	0.83, 1.19	0.97	0.78, 1.20
	Greenness/vegetation	0.97	0.78, 1.20	0.87	0.66, 1.15
	GDP	1.03	0.87, 1.20	1.05	0.86, 1.28
	Climate				
	Temperate	1 (Ref.)		Ref	
	Arid	0.62	0.36, 1.07	0.61	0.32, 1.17
	Tropical	1.15	0.78, 1.70	1.18	0.75, 1.88

All independent variables have been z-transformed using the distribution of all study neighborhoods, except for proximity to coast which is binary (yes/no <1 km from coast) and climate zone (categorical). Estimates in bold font represent estimates with statistical significance of $p<0.05$.

Supplemental Table S3: Multilevel associations between flooding and urban features comparing alternative greenspace metrics in main analysis (NDVI) and sensitivity analysis (greenspace as % of neighborhood land area).

		Main analysis: (greenspace = NDVI)		Sensitivity analysis: (greenspace = % of land area)	
		Estimate	95% CI	Estimate	95% CI
Neighborhood-Level	Population density*	**0.47**	0.41, 0.52	**0.63**	0.56, 0.71
	Education	**0.81**	0.76, 0.86	**0.88**	0.82, 0.94
	Intersection density	**0.71**	0.65, 0.78	**0.81**	0.75, 0.88
	Greenness	**1.43**	1.32, 1.55	**1.24**	1.21, 1.27
	Distance from city center	**1.19**	1.13, 1.25	**1.16**	1.10, 1.22
	Coastal (<1 km)	**46.6**	38.6, 56.3	**51.37**	42.43, 62.19
	Altitude	**1.39**	1.16, 1.66	**1.39**	1.16, 1.67
	Slope	**0.79**	0.75, 0.84	**0.75**	0.70, 0.79
City-level	Population size	0.95	0.83, 1.09	0.95	0.83, 1.09
	Population density	0.93	0.72, 1.19	0.92	0.71, 1.18
	Education	0.95	0.72, 1.25	0.91	0.69, 1.20
	Intersection density	0.99	0.83, 1.19	0.98	0.82, 1.19
	Greenness/vegetation	0.97	0.78, 1.20	0.97	0.78, 1.20
	GDP	1.03	0.87, 1.20	1.02	0.87, 1.20
	Climate				
	Temperate	1 (Ref.)		Ref	
	Arid	0.62	0.36, 1.07	0.66	0.38, 1.14
	Tropical	1.15	0.78, 1.70	1.22	0.82, 1.81

All independent variables have been z-transformed using the distribution of all study neighborhoods, except for greenspace in sensitivity analysis (per 10% higher greenspace as % of neighborhood land area), proximity to coast which is binary (yes/no <1 km from coast) and climate zone (categorical). Estimates in bold font represent estimates with statistical significance of $p<0.05$.

Supplemental Figure S1: Spearman correlation coefficients for covariates in the main model of the associations between neighborhood flooding and features of the urban environment.

	Neighb. educ.	Neighb. int. density	Neighb. green	Neighb. distance	Neighb. coastal	Neighb. elevation	Neighb. slope	City pop. size	City pop. density	City educ.	City int. density	City green	City GDP
Neighb. pop. density	Corr: 0.048	Corr: 0.196	Corr: -0.261	Corr: -0.121	Corr: -0.043	Corr: 0.144	Corr: 0.016	Corr: 0.227	Corr: 0.303	Corr: 0.084	Corr: 0.040	Corr: 0.037	Corr: 0.072
Neighb. educ.		Corr: 0.167	Corr: -0.408	Corr: -0.166	Corr: -0.024	Corr: 0.073	Corr: -0.334	Corr: 0.118	Corr: -0.143	Corr: 0.449	Corr: 0.026	Corr: -0.265	Corr: 0.131
Neighb. int. density			Corr: -0.249	Corr: -0.141	Corr: -0.049	Corr: 0.073	Corr: -0.115	Corr: 0.020	Corr: 0.115	Corr: 0.097	Corr: 0.162	Corr: -0.070	Corr: -0.024
Neighb. green				Corr: 0.200	Corr: 0.062	Corr: -0.116	Corr: 0.357	Corr: -0.191	Corr: 0.103	Corr: -0.255	Corr: -0.174	Corr: 0.572	Corr: -0.236
Neighb. distance					Corr: 0.016	Corr: 0.025	Corr: 0.023	Corr: 0.293	Corr: 0.054	Corr: 0.075	Corr: -0.151	Corr: -0.057	Corr: 0.173
Neighb. coastal						Corr: -0.257	Corr: 0.017	Corr: -0.090	Corr: -0.021	Corr: -0.034	Corr: -0.034	Corr: 0.072	Corr: -0.010
Neighb. elevation							Corr: 0.124	Corr: 0.428	Corr: 0.334	Corr: 0.160	Corr: 0.002	Corr: -0.071	Corr: -0.037
Neighb. slope								Corr: 0.003	Corr: 0.265	Corr: -0.049	Corr: -0.019	Corr: 0.148	Corr: -0.062
City pop. size									Corr: 0.413	Corr: 0.225	Corr: -0.218	Corr: -0.072	Corr: 0.384
City pop. density										Corr: 0.176	Corr: 0.192	Corr: 0.252	Corr: -0.059
City educ.											Corr: 0.135	Corr: -0.347	Corr: 0.298
City int. density												Corr: -0.294	Corr: -0.075
City green													Corr: -0.234

Supplemental Figure S2: Within-city variation in neighborhood flooding in two selected cities: (a) Buenos Aires, Argentina and (b) Medellin, Colombia. Black lines represent neighborhood boundaries and colors represent number of distinct floods from 2000-2018 in the Global Flood Database. The metropolitan area of Buenos Aires, Argentina (left panel), and Medellín, Colombia (right panel).

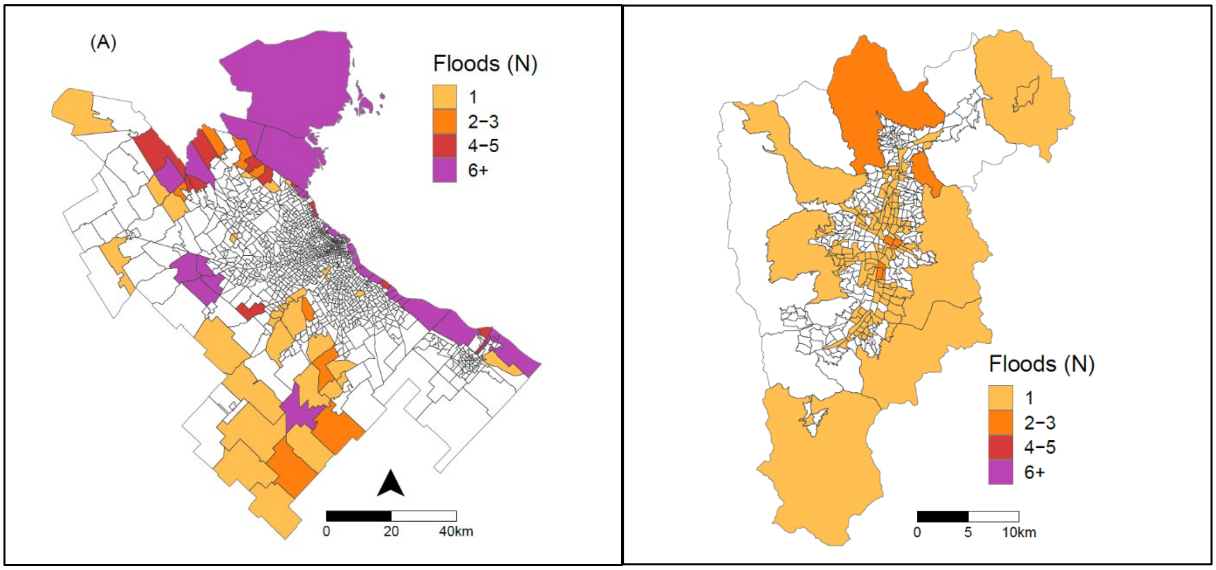

Appendix I: Detailed descriptions of urban environmental measures and their interpretation

Population (neighborhood/city). Number of residents reported by most recent available national census (see Supplemental Table S1 for census year for each country).

Population Density (neighborhood/city). Population (in thousands) per square kilometer of neighborhood/city area. Population sourced from national censuses.

Educational attainment (neighborhood/city). Percent of the population aged 25 years or older who completed primary education or above. Sourced from national censuses.

Intersection Density (neighborhood/city). The number of intersections per area of city/neighborhood built-up area in square kilometers. Intersections were extracted from street network OpenStreetMap data and included any intersections with >2 connected streets (i.e., cul-de-sacs and road bends represented as a node were excluded). Neighborhood and city indices represent the years of 2020 and 2017, respectively.

Greenness (neighborhood/city). Area median greenness measured by the normalized difference vegetation index (NDVI). NDVI was calculated using MODIS satellite-based observations from the MODIS vegetation product, MOD13Q1.006 for 2015 at a 250 m spatial resolution. We computed the maximum NDVI value for 2019 at 250 m resolution to present the 'greenest' condition of each grid cell within the year, then calculated the median across grid cells contained within each neighborhood.

Distance from city center (neighborhood). Euclidean distance in km between the neighborhood centroid and city hall, based on neighborhood boundaries at time of census.

Gross Domestic Product per Capita (city). City-level GDP in 2011 international US Dollars. Created by Genaioli et al in 2013 and converted into gridded estimates by Kummu et al in 2015. GDP for each year 1990-2015 was estimated by these researchers by modeling data from government, survey and industry. Gridded estimates were matched to SALURBAL cities and GDP was extracted directly from matching administrative units or using population-weighted averages in cases where city boundaries crossed multiple administrative areas. GDP estimates from 2015 (most recent year available) were used.

Gennaioli N, La Porta R, Lopez-de-Silanes F, Shleifer A, Human Capital and Regional Development, The Quarterly Journal of Economics, Volume 128, Issue 1, February 2013, Pages 105–164, https://doi.org/10.1093/qje/qjs050.

Kummu M, Taka M, & Guillaume J. Gridded global datasets for Gross Domestic Product and Human Development Index over 1990–2015. Sci Data 5, 180004 (2018). https://doi.org/10.1038/sdata.2018.4.

Coastal Neighborhood. Coastal geographies are those with parts of their boundaries within 1 km from the closest ocean coastline, whereas inland regions represent the rest**.** Coastal lines are obtained from Global Self-consistent, Hierarchical, High-resolution Geography Database (GSHHG) (https://www.ngdc.noaa.gov/mgg/shorelines/, high resolution version).
